# Real-Time Time-Frequency Two-Dimensional Imaging of Ultrafast Transient Signals in Solid-State Organic Materials

**DOI:** 10.3390/s100504253

**Published:** 2010-04-28

**Authors:** Jun Takeda, Akihiro Ishida, Yoshinori Makishima, Ikufumi Katayama

**Affiliations:** 1 Department of Physics, Graduate School of Engineering, Yokohama National University, 79-5 Tokiwadai, Hodogaya, Yokohama 240-8501, Japan; 2 Interdisciplinary Research Center, Yokohama National University, 79-7 Tokiwadai, Hodogaya, Yokohama 240-8501, Japan; E-Mail: katayama@ynu.ac.jp

**Keywords:** femtosecond, imaging, single-shot, pump-probe transient absorption spectroscopy, *β*-carotene, organic materials, biological materials, photodegradation, **PACS** 78.47.J-, 78.67.-n, 33.80.-b, 82.53.Xa

## Abstract

In this review, we demonstrate a real-time time-frequency two-dimensional (2D) pump-probe imaging spectroscopy implemented on a single shot basis applicable to excited-state dynamics in solid-state organic and biological materials. Using this technique, we could successfully map ultrafast time-frequency 2D transient absorption signals of *β*-carotene in solid films with wide temporal and spectral ranges having very short accumulation time of 20 ms per unit frame. The results obtained indicate the high potential of this technique as a powerful and unique spectroscopic tool to observe ultrafast excited-state dynamics of organic and biological materials in solid-state, which undergo rapid photodegradation.

## 1. Introduction

Observation of frequency-resolved transient signals is of great importance in studying photochemical properties of organic and biological materials. The photochemical reactions and excited-state dynamics in liquid-state have been extensively studied via femtosecond pump-probe transient absorption spectroscopy in the last two decades, while those in solid-state are much less explored because of the practical problems such as buildup of reaction products and structural deterioration after irradiation of intense laser pulses. In order to overcome these limitations, single-shot femtosecond spectroscopy has been proposed [1–3]. Although the single-shot techniques can record multiple temporal data points from a single probe pulse at a given wavelength (frequency), they still require many repetitions to obtain full spectral information, which is generally a key to understand ultrafast excited-state dynamics of organic and biological materials.

Recently, we have developed a *time-frequency two-dimensional (2D) pump-probe imaging spectroscopy* implemented on a single shot basis, which enables us to simultaneously map temporal and spectral data points with femtosecond time resolution, and to visualize ultrafast phenomena in real-time [4–7]. Our imaging spectroscopy does not require many repetitions of pump-probe sequence thereby avoiding undesirable effects due to photodegradation or structural deterioration of samples after intense photoirradiation. In addition, since any circulation/rotation systems of samples and a large amount of samples are not needed, it will be a powerful spectroscopic tool to investigate ultrafast excited-state dynamics including irreversible processes in solid-state organic and biological materials.

As demonstrations for our imaging spectroscopy, we measured the ultrafast internal conversions of *β*-carotene in solution and solid films, which play two important roles in photosynthesis: light-harvesting through its singlet states and photoprotection through its triplet states [8,9]. According to the previous studies, the electronic states of carotenoids are in general classified by *C*_2*h*_ symmetry of the polyene backbone. Using this symmetry, the ground state of the carotenoids is expressed as 1^1^*A_g_*^−^ (*S*_0_) state. The lowest singlet excited-state is 2^1^*A_g_*^−^ (*S*_1_) state, which is optically forbidden due to its parity, while the lowest optically allowed excited-state is 1^1^*B_u_*^+^ (*S*_2_) state. On the way of the internal conversion from 1^1^*B_u_*^+^ to 2^1^*A_g_*^−^ state, existence of other forbidden states such as 1^1^*B_u_*^−^ and 3^1^*A_g_*^−^ states has been proposed [10–16]. On the contrary, recently, Kosumi *et al*. show that the ultrafast relaxation process of *β*-carotene can be explained by a three-level model (*S*_0_, *S*_1_ and *S*_2_) without involving any intermediate states [17,18]. Although the assignment of the electronic states is still controversial, here, we simply follow the three-level model to analyze the experimental data.

In this review, we will first describe an overview of the time-frequency 2D pump-probe imaging spectroscopy, and then present a demonstration for the 2D imaging of the ultrafast internal conversion of *β*-carotene in solution. Next, photodegradation processes of *β*-carotene in solid films will be briefly shown. Finally, we will demonstrate a time-frequency 2D imaging applicable to *β*-carotene in solid films. As far as we know, this is the first observation of ultrafast excited-state dynamics of carotenoids in solid-state.

## 2. Real-Time Time-Frequency 2D Pump-Probe Imaging Spectroscopy

In this section, we will present a new spectroscopic tool applicable to visualizing ultrafast photochemical reactions and excited-state dynamics in organic and biological materials–femtosecond real-time time-frequency 2D imaging spectroscopy.

### 2.1. Principle of Femtosecond Real-Time Time-Frequency 2D Imaging Spectroscopy

Real-time time-frequency 2D pump-probe imaging spectroscopy is essentially similar to previously reported single-shot pump-probe technique [2] but uses a white-light continuum as a probe. The basic concept of this technique is as follows; the collimated pump and probe beams separated by an angle *θ* are cylindrically focused such that their focal lines are spatially coincident within a sample, which produce a spatially encoded time-delay for the probe beam. Then the probe beam is spectrally resolved by a monochromator. As the result, both time- and frequency-resolved absorbance changes can be simultaneously obtained.

**Figure 1.**
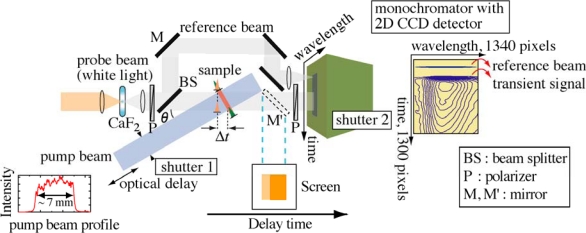
Schematic experimental apparatus for real-time pump-probe imaging spectroscopy implemented on a single shot basis.

Figure 1 shows a schematic experimental apparatus for the real-time time-frequency 2D pump-probe imaging spectroscopy implemented on a single shot basis [4,5]. The fundamental laser pulse from a Ti:sapphire regenerative amplifier system with a repetition rate of 1 kHz, a center wavelength of 800 nm and a pulse duration of 100 fs is divided into two beams; one is used as the pump pulse after second-harmonic generation (400 nm) in a BBO (Type I) crystal, while the other is used to generate a white-light continuum by focusing it on a CaF_2_ thin plate. To avoid thermal heating, the CaF_2_ thin plate is constantly rotated during the measurements and resultantly a stable and intense white-light continuum is obtained in visible and UV light regions [19,20]. The white-light continuum is further divided into two beams by a beam splitter (BS); one is utilized for probe pulse itself, while the other used for reference one to normalize absorbance changes for the same spectral profile. To obtain an excellent time-frequency 2D image of the absorbance changes, one may suppose that normalization for both the same spectral and spatial profiles might be needed. However, since the satisfactory 2D images could be obtained only by the normalization for the same spectral profile (see Figure 4), we conclude that the normalization for the same spectral profile is enough for our measurements [5]. Therefore the reference beam is focused on an entrance slit of a monochromator using a focusing lens.

When performing the imaging experiments on film samples, to avoid photodegradation as much as possible without any sample circulating/rotating systems, we insert a solenoid shutter (shutter 1) having a time response of 10 ms upon the pump beam passage. The pump beam passed through the shutter is magnified to ∼1.2 cm diameter, and then the edge of the beam is clipped by passing through a mask to make a square-shaped pump beam with a size of ∼7 × 7 mm^2^ and a spatially homogeneous intensity. The typical pump beam profile obtained is illustrated in Figure 1. The collimated pump and probe beams intersect with an angle of *θ* = ∼21° and are linearly focused onto a sample with cylindrical lenses and the pump beam is incident normal to the sample. Since the probe beam reaches different parts of the sample at different times, a time-delay between the pump and probe beams is spatially encoded across the sample. After passing through the sample, the probe beam is recollimated and linearly focused on an entrance slit of a monochromator coupled with a 2D charge coupled device (CCD) imaging array detector (1340 × 1300 pixels) and a shutter (shutter 2) having a minimum time response of 8 ms. To remove scattered light from the excitation and fundamental laser pulses, we place appropriate bandpass filters in front of the monochromator. Temporal information of the probe beam is analyzed along the direction parallel to the slit, whereas the spectral information is recorded along the direction normal to the slit. Finally, the real-time 2D imaging of time- and frequency-resolved absorbance changes are obtained. Under our experimental conditions, the time resolution per pixel is 12.5 fs and the whole mapping area per unit frame covers wide spectral and temporal ranges of 420–650 nm and ∼6 ps, respectively.

**Figure 2.**
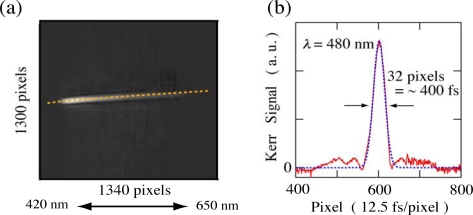
(a) Time-frequency 2D image of the probe beam (white light continuum) measured by the optical Kerr gate method with a 1 mm thickness quartz plate. (b) Temporal evolution of the Kerr signal at 480 nm reproduced from the 2D image of Figure 2(a) [5].

The time resolution of the imaging spectroscopy strongly depends on the sample thickness, the interbeam angle and the laser pulse duration: for our experimental conditions with 1 and 0.25 mm sample thicknesses, the time resolution is estimated to be ∼300 and ∼200 fs, respectively [4,5,21]. The actual time resolution of our imaging method is evaluated from a third-order correlation function for the probe beam measured by the optical Kerr gate (OKG) method [22–25]. The sample is replaced by a quartz plate with 1 or 0.25 mm thickness at the sample position. The two polarizers (P) in Figure 1 are arranged in a crossed configuration and the polarization of the pump beam is set to 45 degree against that of the probe beam. For example, the time-frequency 2D image of the probe beam measured by the OKG method with a 1 mm thickness quartz plate is shown in Figure 2(a). The time evolution of the Kerr signal (third-order correlation function) reproduced from the 2D image is also depicted in Figure 2(b). Judging from the full width of half maximum (FWHM) of the Kerr signal, the time resolution of the imaging spectroscopy is less than ∼400 fs in whole spectral range from 420 to 650 nm, whose value is comparable with the calculated one. Similarly, for the sample thickness of 0.25 mm, the time resolution is estimated to be less than ∼300 fs in the whole spectral range. The 2D image of the Kerr signal also shows a chirp characteristic due to group velocity dispersion (GVD) of the probe beam as shown by a dotted line in Figure 2(a). This 2D image is stored and used to correct the GVD of actual pump-probe imaging data. The spatial intensity profile of the pump beam with a nearly homogeneous intensity is also recorded by a CCD detector and used to normalize the transient absorption change on a personal computer.

### 2.2. Time-Frequency 2D Imaging of Ultrafast Transient Absorption of β-Carotene in Solution

In order to confirm that our time-frequency 2D pump-probe imaging spectroscopy really works well to observe the ultrafast excited-state dynamics in organic and biological materials, we measured the ultrafast internal conversion of *β*-carotene in solution, which has been extensively studied by the conventional pump-probe spectroscopy [10–18]. The *β*-carotene was dissolved in *n*-hexane with a concentration of 1.4 × 10^−4^ M (1 M = 1 mol/dm^3^). The solution is circulated by a peristaltic pump through a flow cell with 1 mm path length during the measurements, to avoid thermal heating of the samples. The excitation power and area of the pump pulse at the sample are 20 μJ and 6.5 × 0.2 mm^2^, respectively, resulting a photon density of ∼3 × 10^15^ photons/cm^2^.

**Figure 3.**
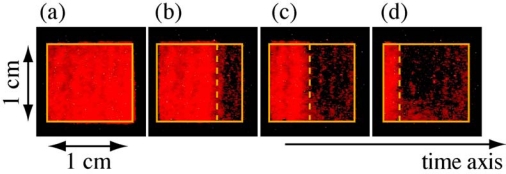
Images of the probe beam transmitted through *β*-carotene solution (a) with the pump beam off and the pump beam on with different delay times of (b) 0, (c) 1 and (d) 2 ps, respectively. To clearly visualize the transient absorption image on the screen, we made *β*-carotene solution with a high concentration (∼1 × 10^−3^ M).

Before measuring the frequency-resolved 2D image of the ultrafast internal conversion using a monochromator with a 2D CCD detector, in order to ensure that the probed beam passed through the sample, we inserted a mirror (M') in front of the monochromator and imaged the probe beam on a 1 × 1 cm^2^ screen (see Figure 1). Since only visible light part of the probe beam is transmitted through the sample, the image contains information of the transient absorption of *β*-carotene. The image was taken by a digital camera with an exposure time of 1 s. Figure 3 shows the images of the transmitted probe beam (a) with the pump beam off, and the pump beam on with different delay times, (b) 0, (c) 1 and (d) 2 ps, respectively. The delay between the pump and probe beams is tuned by a mechanical translation stage (optical delay). In the images, the time-encoding direction is horizontal. When the pump beam is blocked, the image (a) shows a homogeneous color (orange) that reflects the transmission of the probe without any transient absorption of *β*-carotene. When the pump beam is on, on the other hand, the images (b)–(d) have a dark area that moves along the time-encoding direction with the delay. This dark area corresponds to the transient absorption of *β*-carotene, which attenuates the transmission intensity of the probe. This result strongly shows that our technique successfully works to visualize the ultrafast transient absorption of organic materials with a wide temporal range of 5–6 ps in real-time.

**Figure 4.**
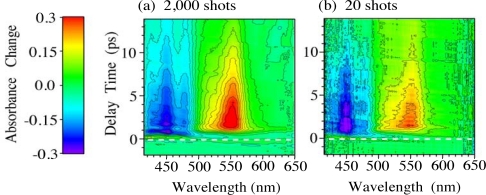
Time-frequency 2D image of transient absorbance changes of *β*-carotene in *n*-hexane solution mapped by the time-frequency 2D pump-probe imaging spectroscopy with accumulations of (a) 2,000 and (b) 20 laser shots [5].

Next, we demonstrate the real-time time-frequency imaging of the ultrafast internal conversion of *β*-carotene in solution. Figure 4 shows the time-frequency 2D images of transient absorbance changes of *β*-carotene in *n*-hexane solution with accumulation times of (a) 2 s (2,000 laser shots) and (b) 20 ms (20 laser shots) per unit frame. These images are obtained after corrections of the GVD and of the spatial profile of the pump pulses [5]. The intensity of the absorbance changes is indicated as contours. The 2D images are obtained by adding several frames with different delay times in order to cover the entire relaxation process of *β*-carotene. Since the absorbance changes are normalized for the same spectral profile at each frame (see Figure 1), we can obtain a satisfactory 2D image of the absorbance changes [5].

**Figure 5.**
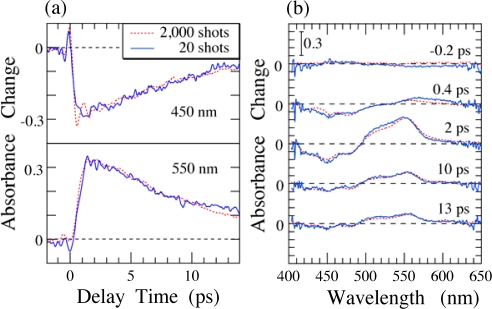
(a) Temporal evolution of the absorbance changes at 450 and 550 nm, and (b) time-resolved absorbance change spectra with different delay times reproduced by the 2D images shown in Figure 4 [5].

Figure 5 shows (a) time evolution of the absorbance changes and (b) time-resolved absorbance change spectra with different delay times reproduced by the 2D images shown in Figure 4. The 2D images clearly show the instantaneous absorption bleaching of 1^1^*A_g_*^−^ state at 420–500 nm and the transient absorption from 2^1^*A_g_*^−^ to higher states at 500–620 nm with rise and decay times of ∼1 and ∼10 ps, respectively [10,26]. Although the spectra obtained by the accumulation of 20 laser shots (solid lines) are slightly noisy, not only the intensity but also the spectral shape is in quite good agreement with those obtained by the accumulation of 2,000 laser shots. This indicates that our time-frequency 2D pump-probe imaging spectroscopy really works well even with very short accumulation time, suggesting that this technique would be a unique and powerful spectroscopic tool applicable to observation of photochemical reactions and excited-state dynamics in organic and biological materials, which easily undergo photodeterioration after irradiation of intense laser pulses. Actually, using this technique, very recently, we could also successfully measure the time-frequency 2D image of the transient absorbance changes of light-harvesting π-conjugated dendrimers in solution even with tiny small amount of the samples [27,28].

## 3. Photodegradation Processes of *β*-Carotene in Solid Films

### 3.1. Photobleaching for Steady-State Absorption Spectra

As shown in the former section, using our imaging spectroscopy, the time-frequency 2D images of ultrafast excited-state dynamics in organic and biological materials can be measured with very short accumulation time based on a single-shot detection. The results strongly demonstrate the high potential of the imaging spectroscopy to detect photochemical reactions and excited-state dynamics in solid-state which easily undergo photodegradation and structural deterioration. Here, we will present the results on strong photodegradation processes of *β*-carotene in solid films, which is undesirable for measuring ultrafast transient signals with many repetitions of pump-probe sequence.

For this study, commercial powders of all-*trans-β*-carotene (special grade) were used without further purification. Poly(methylmethacrylate) (PMMA) was used as a host matrix. *β*-carotene was dispersed in PMMA film with a concentration of 1.0 × 10^−3^ M by the following method; *β*-carotene powders and PMMA beads were diluted with dichloromethane. Then, the mixed solution was slowly dried in a flat schale with a 70 mm diameter. The typical thickness of the film is 250 μm. The film obtained was cut out with 1 × 1 cm^2^ area to make a film sample. The film sample with 1 × 1 cm^2^ area contains only 18 μg of *β*-carotene, whose quantity is two or three orders of magnitude less than that used typically for the conventional pump-probe transient absorption spectroscopy.

**Figure 6.**
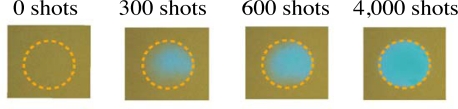
Photodegradation of *β*-carotene in PMMA film with different numbers of laser shots. The excitation photon density is 3.0 × 10^15^ photons/cm^2^.

To investigate the photodegradation process of *β*-carotene in PMMA films, we use second harmonic pulses (400 nm) from a Ti:sapphire regenerative amplifier system with a repetition rate of 1 kHz and a pulse duration of 100 fs as the excitation laser pulses. The excitation laser pulses were irradiated into a film sample with different numbers of laser pulses and photon densities. With irradiation of the excitation laser pulses, the color of the film immediately changes from yellow to transparency, implying that a permanent photodegradation of *β*-carotene in PMMA films takes place (see Figure 6).

**Figure 7.**
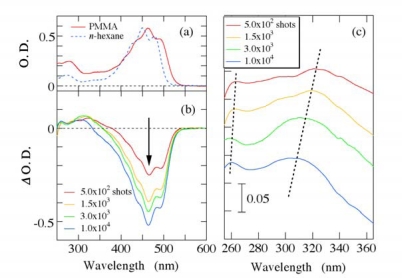
(a) Steady-state absorption spectrum of *β*-carotene in PMMA films, and (b) its absorbance change spectra after photoirradiation of the excitation laser pulses with an excitation density of 3 × 10^15^ photons/cm^2^. The steady-state absorption spectrum of *β*-carotene in *n*-hexane solution with a concentration of 1 × 10^−4^ M is also indicated by a broken line. (c) The absorbance change spectra in UV light region are depicted on an appropriate enlarged scale with different offsets on y-axis.

In order to evaluate the photodegradation process quantitatively, the steady-state absorption spectra of the film sample were measured before and after irradiation of the excitation laser pulses by using a conventional UV-VIS spectrometer. Figure 7 shows (a) steady-state absorption spectrum of *β*-carotene in PMMA film and (b–c) its absorbance changes (*Δ* O. D. = *Δ* optical density) after irradiation of the pump laser pulses with an excitation density of 3 × 10^15^ photons/cm^2^. As a comparison, the steady-state absorption spectrum of *β*-carotene in *n*-hexane solution with a concentration of 1 × 10^−4^ M is also indicated by a broken line in Figure 7(a). The energy position of the fundamental absorption band of *β*-carotene in PMMA films is lower than that in *n*-hexane solution due to polarizability of the solvent/matrix [29]. Except for the ∼15 nm red-shift and a slight spectral broadening, the steady-state absorption spectrum of *β*-carotene in PMMA films looks essentially the same as that in *n*-hexane solution. After irradiation of a few thousand pump laser shots (a few seconds), the absorption band at 400–550 nm, which corresponds to the optical transition from 1^1^*A_g_*^−^ to 1^1^*B_u_*^+^ state, decreases due to strong photodegradation. On the contrary, the absorbance change in UV light region slightly increases having a blue-shift with irradiation of the excitation laser pulses as shown by broken lines in Figure 7(c).

**Figure 8.**
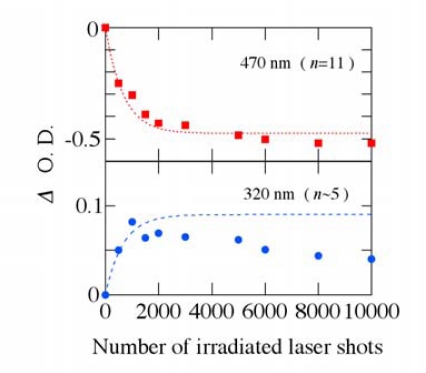
Absorbance changes due to the photodegradation of *β*-carotene in PMMA films as a function of number of the irradiated excitation laser shots.

Figure 8 shows the absorbance changes at 470 and 320 nm due to the photodegradation as a function of number of the irradiated excitation laser shots. As number of the irradiated laser pulses increases, the absorption intensity at 470 nm decreases and eventually the absorption due to the optical transition from 1^1^*A_g_*^−^ to 1^1^*B_u_*^+^ state disappears. On the other hand, the absorption intensity around 320 nm first increases and then gradually decreases with increasing the irradiated laser shots.

It is well known that energy position on the fundamental absorption band in π-conjugated polyenes becomes higher with decreasing the conjugation length (number of the conjugated double bonds *n*); the number *n* is empirically proportional to a square of the center wavelength of the absorption band [30,31]. According to this empirical rule, the fundamental absorption band of *β*-carotene (*n* = 11) lies at ∼470 nm, while that of the carotenoids with *n*∼5 is located around ∼320 nm. Judging from the experimental results and the empirical rule, the linear polyene structure of all-*trans*-*β*-carotene might be broken into shorter polyene segments and/or partly bent with irradiation of intense pump laser pulses, leading to number of the conjugated double bonds *n* being smaller and the effective conjugation length becoming shorter from *n* = 11 to *n*∼5. Subsequently, it may be further shortened. Because the strong absorbance due to PMMA matrix itself lies below 250 nm, a further shortening process on the linear polyene structure of *β*-carotene could not be observed in UV light region below 250 nm.

### 3.2. Possible Mechanism for the Photodegradation of β-Carotene in Solid Films

Assuming that *β*-carotene in PMMA films becomes photodegraded in proportion to number of remaining *β*-carotene molecules with irradiation of the pump laser shots, the decrease of the absorbance change at 470 nm is expressed as −*A*(1−*e*^−*γN*^), where *N* is the number of pump laser shots, *γ* is a photodegradation coefficient and *A* is a constant (*A* > 0). Similarly, the increase of the absorbance change around ∼320 nm due to the photoproducts, which come from dissociation of *β*-carotene into the shorter π-conjugated segments, might be given by *B*(1−*e*^−*γN*^) (*B* > 0). The broken lines in Figure 8 show the best fit by this procedure with *γ* = 1.5 × 10^−3^/shot, and the experimental results can be well explained by this simple model. Because the photoproducts might be further photodegraded to those having smaller *n*, the absorbance change around ∼320 nm gradually decreases after the irradiation of ∼2,000 laser shots.

**Figure 9.**
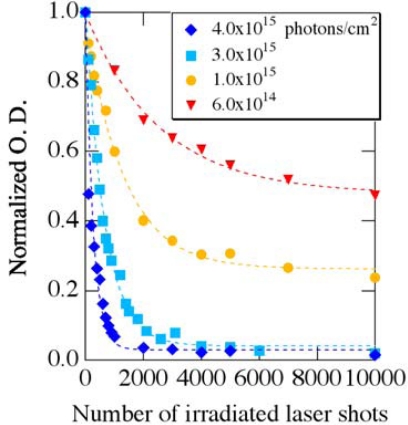
Photobleaching of the normalized absorbance at 470 nm as a function of number of the irradiated excitation laser shots with different photon densities.

Figure 9 shows photobleaching of the normalized absorbance at 470 nm as a function of the irradiated laser shots with different excitation photon densities. Under the weak excitations (< ∼1.0 × 10^15^ photons/cm^2^), the normalized absorbance intensity decreases with the irradiated laser shots, and then it is saturated. On the other hand, the photobleaching process completely takes place above ∼3.0 × 10^15^ photons/cm^2^. This suggests that some fraction of *β*-carotene molecules in PMMA films survives without photodegradation under the weak excitations. The similar behavior was observed for temperature dependence on photochemical reactions of functional organic materials in polymer films [32–34]: all of the molecules undergo photochemical reactions at high temperature, while some fraction of molecules does not at low temperature. This behavior is considered to come from the distribution of local free volume in glassy polymers, and the limitation in the freedom of molecular motion usually leads to the non first-order process of the reactions. The same situation might occur in the photobleaching process of *β*-carotene molecules in PMMA films. Except for the non-photodegraded molecules under the weak excitations, however, the photobleaching process is well fitted by the above simple model as shown by broken lines.

**Figure 10.**
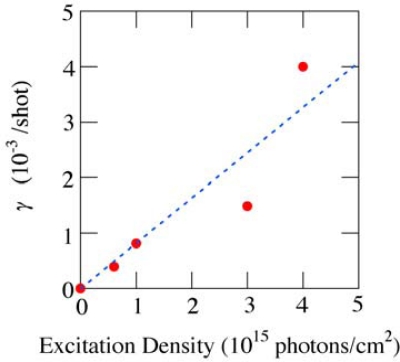
Photodegradation coefficient *γ* as a function of the irradiated excitation density.

The photodegradation coefficient *γ* as a function of the excitation density is also shown in Figure 10. Since the coefficient *γ* is proportional to the excitation density as shown by a broken line, our simple model seems reasonable as a model of the photodegradation process of *β*-carotene. Since this process is in linear regime, the photodegradation might occur even if the excitation density is much reduced. Therefore, femtosecond pump-probe laser spectroscopy based on a single-shot detection is highly desired to measure ultrafast excited-state dynamics of carotenoids in solid-state thereby avoiding the photodegradation effects.

## 4. Time-Frequency 2D Imaging of Transient Absorption of *β*-Carotene in Solid Films

As mentioned in the above section, with irradiation of femtosecond pump laser pulses, the linear polyene structure of *β*-carotene in PMMA films is most likely broken into shorter polyene segments and/or is partly bent. As the results, *β*-carotene in solid films shows rapid photodegradation within a few seconds. Because of this limitation, the conventional pump-probe absorption technique is not feasible for observation of ultrafast excited-state dynamics in solid films. On the contrary, here, we demonstrate that our imaging spectroscopy enables us to measure the real transient signals in solid-state organic materials against any backgrounds due to undesirable photoproducts.

**Figure 11.**
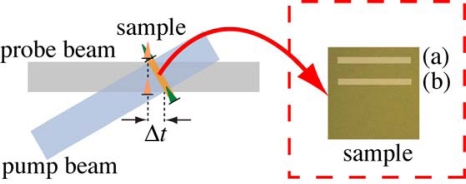
Schematic optical apparatus around the sample position for the time-frequency 2D pump-probe imaging spectroscopy applicable to solid-state samples.

After the irradiation of 2,000 pump laser shots (2 seconds) with an excitation density of 3 × 10^15^ photons/cm^2^, the steady-state absorption of *β*-carotene in PMMA films disappears almost completely due to the strong photodegradation (see Figures 7 and 8). After the irradiation of 20 pump laser shots, on the other hand, since 95% of *β*-carotene in PMMA films remains without the photodegradation, we are able to observe the ultrafast transient signals of *β*-carotene in PMMA films using our imaging spectroscopy. When performing the imaging experiments, the timing between the shutters 1 and 2 (Figure 1) is controlled by a digital delay generator; the shutter 2 is open during the accumulation of data storage (typically 20 ms), while the shutter 1 is open 5 ms earlier and closed 10 ms later than the shutter 2, keeping to minimize the photodegradation of film samples. Then, as shown in Figure 11, we first measure the time-frequency 2D image of the transient absorption of *β*-carotene in PMMA films with *Δt* = 0 at some area (strip (a)), which contains both real transient signals and undesirable ones due to the photodegradation. Next, we measure the time-frequency 2D image with *Δt* = −100 ps at an area different from the strip (a) (strip (b)), which only contains the undesirable signals. The imaging data obtained by subtracting the data at the strip (b) from those at the strip (a) leave only the real transient signals.

**Figure 12.**
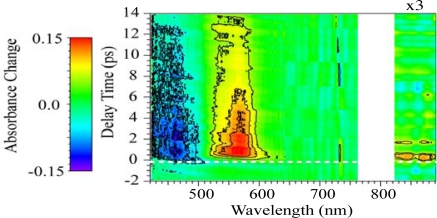
Time-frequency 2D image of the transient absorbance change of *β*-carotene in PMMA films with an accumulation of 20 laser shots per unit frame. The absorbance change is indicated by contours. The intensity of the image between 820 and 890 nm is magnified by three times.

Figure 12 shows the time-frequency 2D image of the transient absorbance change of *β*-carotene in PMMA film with an accumulation of 20 laser shots (20ms) per unit frame obtained by the above procedure. The absorbance change is indicated by contours. The 2D image is made by adding about dozen frames with different delay times and wavelengths to cover the entire transient behavior of *β*-carotene in PMMA films. Due to the strong scattered light from fundamental laser pulses, which are used to generate a white-light continuum, the transient signals between 760 and 820 nm could not be observed. The 2D image clearly shows the instantaneous absorption bleaching at 430–490 nm and the transient absorption at 820–890 nm and at 520–600 nm with a fast rise time (see a broken line in Figure 12).

**Figure 13.**
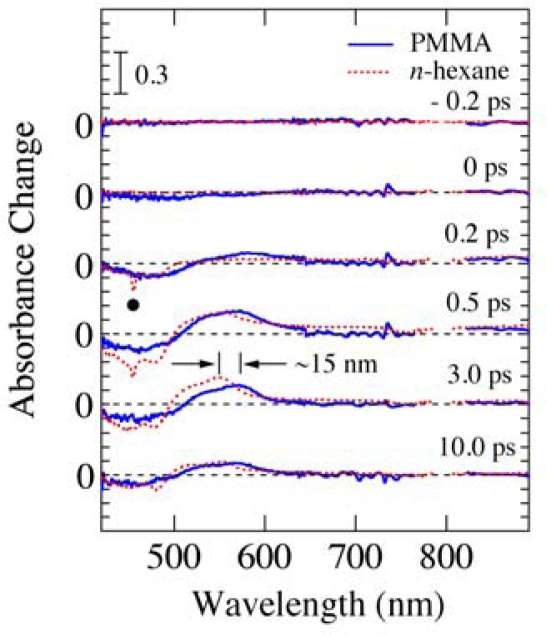
Time-resolved absorbance change spectra of *β*-carotene in PMMA films with different delay times. As comparison, the spectra of *β*-carotene in *n*-hexane solution measured by the conventional pump-probe transient absorption spectroscopy are also shown by dotted lines.

Figure 13 shows the time-resolved absorbance change spectra with different delay times reproduced by slicing off the 2D image shown in Figure 12. As comparison, the experimental data of *β*-carotene in *n*-hexane solution measured by the conventional pump-probe transient absorption spectroscopy are also shown by dotted lines. A sharp negative peak due to the Raman gain (a closed circle) is observed at 455 nm for *β*-carotene in *n*-hexane solution [35,36], but not observed for *β*-carotene in PMMA films. The Raman gain observed has a shift of ∼3000 cm^−1^, which is mainly due to the C-H stretching mode of *n*-hexane. Therefore, it is not observed in *β*-carotene in PMMA films. In addition, there is a red-shift of ∼15 nm between the transient absorption spectra in PMMA film and in *n*-hexane solution, which is also seen in the steady-state absorption spectra (see Figure 7(a)). Except for these two points, however, the observed transient behavior of *β*-carotene in PMMA film seems essentially the same as that in *n*-hexane solution.

**Figure 14.**
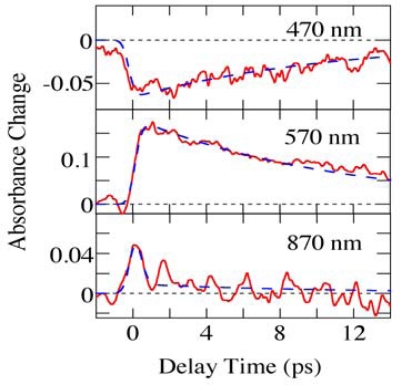
Time evolution of the absorbance changes of *β*-carotene in PMMA films with different wavelengths. The fitted curves calculated from assumption of the three-level system are also shown in broken lines.

Figure 14 shows the time evolution of the absorbance changes of *β*-carotene in PMMA films with different wavelengths reproduced from the 2D image of Figure 12. Although the data at 870 nm show poor signal to noise ratio, not only the absorption bleaching at 470 nm but also the transient absorption lying at visible and infrared regions is observed. The electronic states of *β*-carotene have been studied based on three-level or four-level systems. Here, we analyze the ultrafast transient absorption data of *β*-carotene in PMMA films based on the three-level system involving 1^1^*A_g_*^−^, 1^1^*B_u_*^+^ and 2^1^*A_g_*^−^ states [17,18]; the absorption bleaching of 1^1^*A_g_*^−^ state, the transition from the 1^1^*B_u_*^+^ to higher excited states, the transition from 2^1^*A_g_*^−^ to higher excited states via the internal conversion from 1^1^*B_u_*^+^ to 2^1^*A_g_*^−^ state, and the recovery from 2^1^*A_g_*^−^ to 1^1^*A_g_*^−^ state are taken into account. The broken lines at 470 and 570 nm show the best fitted ones convoluted with the system response function under the assumption of the three-level system. Also, using the same time constants, the time profile of the absorbance change at 870 nm is roughly reproduced. The time constants obtained are listed in Table I together with those previously reported in different kinds of solutions. As shown in Table 1, the time constants obtained in PMMA films are comparable with those in solutions.

Because our imaging spectroscopy is implemented on a single-shot basis and does not require many repetitions of pump-probe sequence, we can successfully map the time-frequency 2D image of the transient absorption of *β*-carotene in solid films with wide temporal and spectral ranges.

## 5. Conclusions

In conclusion, we demonstrate a real-time time-frequency 2D pump-probe imaging spectroscopy implemented on a single shot basis in femtosecond time regime. To obtain clear time-frequency 2D images with a good signal to noise ratio, not only probe beam but also reference one is simultaneously recorded by a 2D CCD detector. Using this technique, the accumulation time to record time-frequency 2D image of the transient absorbance changes becomes dramatically reduced. Therefore, we can successfully map the time- and frequency-resolved absorbance changes of *β*-carotene in solid films having very short accumulation of 20 laser shots per frame without any sample flowing or rotating system. Since our imaging technique does not require many repetitions of pump-probe sequence and resultantly only small quantities of samples are needed, we believe that this technique is useful for studying solid-state photochemical reaction dynamics in various kinds of organic and biological materials, for which large quantities are not readily available and/or photodegradation process readily takes place.

## Figures and Tables

**Table 1.** The estimated time constants of the internal conversion from 1^1^*B_u_*^+^ to 2^1^*A_g_*^−^ state and the recovery from 2^1^*A_g_*^−^ to 1^1^*A_g_*^−^ state based on the three-level system. As comparison, the values in different solvents are also listed.

solvents/matrix	**Time constant (ps)**	
	internal conversion 1^1^*B_u_*^+^ → 2^1^*A_g_*^−^	recovery 2^1^*A_g_*^−^ → 1^1^*A_g_*^−^
PMMA^*1)^	0.17	10.0
*n*-hexane	0.22^*2)^, 0.28^*3)^	9.4^*2)^, 9.3^*3)^
benzene^*2)^	0.25	9.1
chloroform^*3)^	0.20	9.8

*1) this work,

*2) ref. 18,

*3) ref. 10
